# Editorial Comment: Novel semirigid ureterorenoscope with irrigation and vacuum suction system: introduction and initial experience for management of upper urinary calculi

**DOI:** 10.1590/S1677-5538.IBJU.2019.0521.1

**Published:** 2020-07-31

**Authors:** Eduardo Mazzucchi

**Affiliations:** 1 Faculdade de Medicina de São Paulo da Universidade de São Paulo Divisão de Urologia Departamento de Endourologia SP Brasil Departamento de Endourologia, Divisão de Urologia, Faculdade de Medicina de São Paulo da Universidade de São Paulo, SP, Brasil

## COMMENT

The authors report their experience with a new semirigid scope attached to an irrigation and suction system with the aim of reducing residual fragments and improving the stone-free rates after ureteroscopy for upper ureteral and renal stones (1).

It is well-known by endourologists that elevated intra-renal pressure during the procedure and residual fragments are two of the main problems of ureteroscopy, once they can lead to sepsis and to asymptomatic residual fragments that result in unexpected visits to the emergency room, re-admittances to the hospital and re-interventions in up to 29 % of the cases (2). Therefore, a search for effective suctioning systems attached to the ureteral access sheath in order to reduce intrapelvic pressure during the procedure and the occurrence of residual fragments after breaking the stone has been reported in the literature (3).

Herein the authors report their experience with a suctioning system coupled to a metallic 40 cm long and 11.6/12.9 Fr diameter access sheath and use a 46 cm and 4/6 Fr mini-ureteroscope inside to treat upper ureteral, pelvic, upper and medium calyceal kidney stones. They treated 58 patients with an 89.7% stone-free rate at 1st PO at CT and 1.7% complication rate. The access sheath could not be inserted in only one patient. Interestingly, the stone was located in the mid-calyx in 19% of the cases and the authors did not report any difficulty or failure in reaching out the calyx with a semirigid device inside a metallic access sheath which is recognizably a difficult task.

Other articles evaluating suctioning access sheaths for big upper ureteral stones have been published showing better stone-free rates when compared to the traditional ones (4).

Suctioning systems seems to be in the near horizon in ureteroscopy but this is an initial experience with a small number of cases performed in a single center. It has to pass the test of time to be incorporated in the daily urological practice.
